# Mechanical analysis and failure modes prediction of composite rock under uniaxial compression

**DOI:** 10.1038/s41598-021-02331-x

**Published:** 2021-11-24

**Authors:** Jianguang Li, Zhuoqun Yu, Ziyi Zhou, Yanchun Wang, Jiwei Li

**Affiliations:** School of Mechanical and Electrical Engineering, Qingdao University of Science and Technology, Qingdao, 266061 China

**Keywords:** Structural materials, Civil engineering

## Abstract

Composite rocks are easily encountered in a wide range of geotechnical construction projects. Understanding their mechanical properties and failure modes is very important to ensure project quality and safety. This study conducted a mechanical analysis to assess the stress distribution in composite rock with a horizontal interlayer and predicted the possible failure modes. Uniaxial compression tests were carried out on the composite rock samples to reveal their mechanical properties. It was concluded that a composite rock with a thick interlayer failed more easily than a composite rock with a thin interlayer. Four potential failure modes were related to the internal stress distribution under compression and the differences in deformation capacity and strength among the constituent components. The stress distribution derived from the mechanical analysis could explain the failure mechanism very well. These results verified the validity of the mechanical analysis results and improved understanding of the mechanical properties of composite rock with a horizontal interlayer.

## Introduction

Geotechnical engineering has been developing rapidly, and an increasing number of infrastructure projects involving stratified rock engineering are being carried out in conjunction with China's economic stimulus initiatives and long-term strategic planning^1^. At the same time, with the increasing depletion of global fossil energy sources and the rapid development of guidance technology, an increasing number of underground extraction projects and national defence projects have shifted from shallow to deeper excavations, which have presented rock mechanics problems that have become the focus of engineering design^2^. Varieties of rock failure modes, such as rock bursts, roof falls, and landslides, are often encountered during the service life of a structure^3^. Excluding extreme "natural" factors, most of these failures are related to "man-made" disasters, such as improper planning and design, construction irregularities and poor maintenance measures^2,4,5^. Therefore, it is important to understand the mechanical behaviour and failure modes of various rock types, including single rocks and flawed rocks^6–11^. In deep underground engineering, the chambers, free faces, and pillars would under uniaxial compression and required significant attention. Uniaxial compressive strength (UCS) is the most widely used mechanical property parameter for rocks, and uniaxial compression tests are comparably accessible and cost-effective^12–14^. Mousavi et al.^15^ conducted uniaxial compression tests to study the UCS of schist rock under a freezing–thawing process. They reported that the UCS of schist rock decreased with an increasing number of freeze–thaw cycles. Jalali et al.^16^ estimated the UCS of sedimentary rock by employing prediction models. It was found that the adaptive neuro-fuzzy inference system had the best performance in the prediction of sedimentary rock UCS. Image analysis and acoustic emission analysis are often important tools for studying rock failure^17,18^. For instance, Zhang et al.^19^ used an acoustic monitoring technique to detect the failure processes of flawed sandstone specimens. They reported that the catastrophic rupture of brittle rocks could be forecasted based on precursory acoustic emission time series.

However, the study of single rocks alone is insufficient, as composite rocks containing interlayers are often encountered in practical engineering^20^. For example, sedimentary rocks with stratified structures account for approximately 66.7% of the world's land area and 77.3% in China. The most widely distributed examples are sandstones, claystone, and limestone, which account for approximately 98% of sedimentary rocks. Even many metamorphic rocks can be characterized by laminated structures^21^. When the thickness of certain rocks in a laminated composite rock body is thin and the strength is weak, a composite rock body with a weak interlayer is formed. Special attention is needed for cavern engineering in such rock bodies. The Longkou water conservancy project was a large water conservancy and hydropower project that faced the challenges of near-horizontal weak interlayers in the rock base. Practice has shown that a weak interlayer distributed in a cavity surrounding rock is unfavourable to the overall stability of the cavity surrounding a rock body and the safety of the support structure, which restricts the occurrence and development process of the deformation and failure of an entire project's rock body. Researchers and engineers need to understand the mechanical properties and failure modes of composite rock that contains interlayers^22–24^. Cheng et al.^25^ prepared composite rock specimens with different dip angles using rock-like materials and carried out uniaxial compression tests. The deformation behaviour of composite rock specimens was investigated with the help of a 3D digital image correlation technique. They found that the UCS and failure modes of a composite rock with interlayers were significantly affected by the change in dip angles. Ren et al.^26^ studied the effect of weak interlayer dip angles and anchorage angles on the UCS and failure modes of anchored rock with weak interlayers. Ma et al.^27^ conducted uniaxial compression tests of coal-rock composite specimens with different height ratios using particle flow code (PFC) software. They reported that a smaller coal-rock height resulted in a higher elastic modulus and higher UCS. Chen et al.^28^ investigated the mechanical properties of oil shale-coal composite rock samples by using uniaxial compression tests. It can be seen that the numerical simulation has been carried out widely to explore the failure behaviours of composite rocks due to its intuitive nature. However, there have been few mechanical modelling analyses and experimental studies on composite rocks under the influence of the interlayer thickness and interlayer strength. It is valuable to study the stress distribution inside and outside the interface of composite rocks so that the structural failure mechanism of composite rock masses can be determined from a mechanical point of view.

In this paper, composite rock specimens containing two types of interlayers with six different thicknesses were prepared. A mechanical modelling analysis was carried out to estimate the stress distribution and failure modes of the composite rock specimens. Uniaxial compression tests were conducted to study the mechanical properties and validate the prediction results. The mechanical analysis and experimental methods in this study were intended to provide a reference for predicting failure modes of composite rock masses with different thicknesses of interlayers, so that individualized regulation or targeted support can be provided to specific composite rock masses based on the predicted failure modes. Compared with the numerical simulation analysis which has been widely carried out, the mechanical modelling analysis can help researchers better understand the rock failure principle. At the same time, the results of mechanical analysis are analytical solutions rather than numerical solutions, which have higher adaptability and good generalization. On the other hand, conducting mechanical experiments can provide results that are closer to a real situation and can contrast with a mechanical modelling analysis. Based on the above premises, the innovations of this paper can be described as follows: (i) a mechanical modelling analysis approach was used to derive an analytical solution of the stress distribution and to predict the failure modes of composite rocks; (ii) composite rock specimens with different thicknesses of horizontal interlayers were prepared and assessed by using simulated rock materials; and (iii) failure modes derived from the experimental method were compared with those derived from the mechanical modelling analysis approach.

## Specimen manufacture and experiment procedure

### Materials

Due to the inherent inhomogeneity and discontinuity of real rock masses, the experimental rock specimens obtained at the engineering site have unavoidable dispersion and randomness. It is difficult to freely select the thickness. Therefore, rock-like materials are often used instead of real rocks in many basic theoretical studies aimed at exploring scientific laws^22,24,26^. In this study, rock-like materials were selected according to the results of pre-experiments and accumulated experience from previous studies^25,26^. Ordinary Portland cement 32.5R and gypsum were used as the stronger binder and weaker binder, respectively. River sand smaller than 20 mesh was utilized as the aggregate. Construction glue #801 was used as the retarder to delay the solidification time of gypsum mortar. Tap water was used to prepare the specimen, and the water dosage of the specimen varied from 10 to 12.5% of the mass of the specimen to ensure the good workability of the mortar.

**Figure 1:**
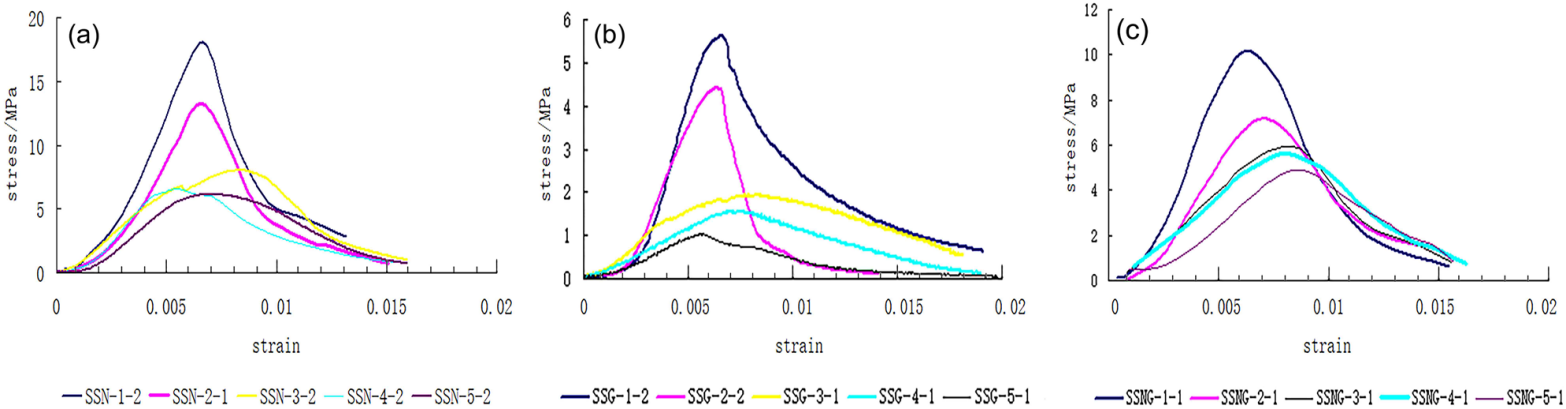
Stress–strain curves of (**a**) SSN, (**b**) SSG, and (**c**) SSNG rock-like materials.

Based on adequate preliminary experiments, appropriate proportions of materials can be found to simulate real rocks. Figure 1 shows the stress–strain curves of the specimens with different mixing ratios of sand, cement and gypsum. The designation of specimens represents the constituent materials and their ratios. The letters SSN, SSG, and SSNG represent that the rock-like materials are sand mixed with cement, sand mixed with gypsum, and sand mixed with cement and gypsum, respectively. The numbers represent the sand-binder ratio (if the cementitious agent is cement and gypsum, the two are mixed by the same mass). For example, “SSN-2-1” represents a specimen made of sand and cement mixed in a ratio of 2:1. After detailed comparisons, SSN-2-1 was chosen to simulate a limestone rock mass, SSG-2-1 was chosen to simulate a shale interlayer, and SSNG-2-1 was chosen to simulate a sandstone interlayer. The mechanical parameters of simulated limestone, shale and sandstone compared with real limestone, shale and sandstone are shown in Table 1. The simulated rock materials had similar mechanical parameters to the real rocks.

**Table 1:** Main mechanical parameters of rock-like materials and real rocks^29^.

Specimens	Unconfined compressive Strength (MPa)	Elastic modulus (GPa)	Poisson’s ratio
Shale	2.17–5.44	0.98–2.51	0.09–0.35
Simulated shale	4.64	1.18	0.29
Sandstone	6.53–10.9	1.09–3.38	0.05–0.36
Simulated sandstone	7.20	1.53	0.25
Limestone	8.72–15.22	2.61–7.62	0.18–0.35
Simulated limestone	13.15	3.13	0.2

### Specimens preparation

Composite rock specimens were prepared using a cylindrical mould 50 mm in diameter and 100 mm in height. The materials were weighed on an electric scale with an accuracy of 0.1 g, and then the weighed materials were mixed evenly in a laboratory mixer with appropriate amounts of water. For the specimen moulding production, the mixture of the main rock mass was placed in the bottom of the cylindrical mould first. Then, the mixture of the interlayer was placed on the top of the main rock mass mixture. Finally, the main rock mass mixture was placed on the top of the interlayer mixture. The height of each part of the composite rock was adjusted by controlling the amount of the mixture. The specimen was cured for 28 days in a humidity chamber before the mechanical test.

**Figure 2:**
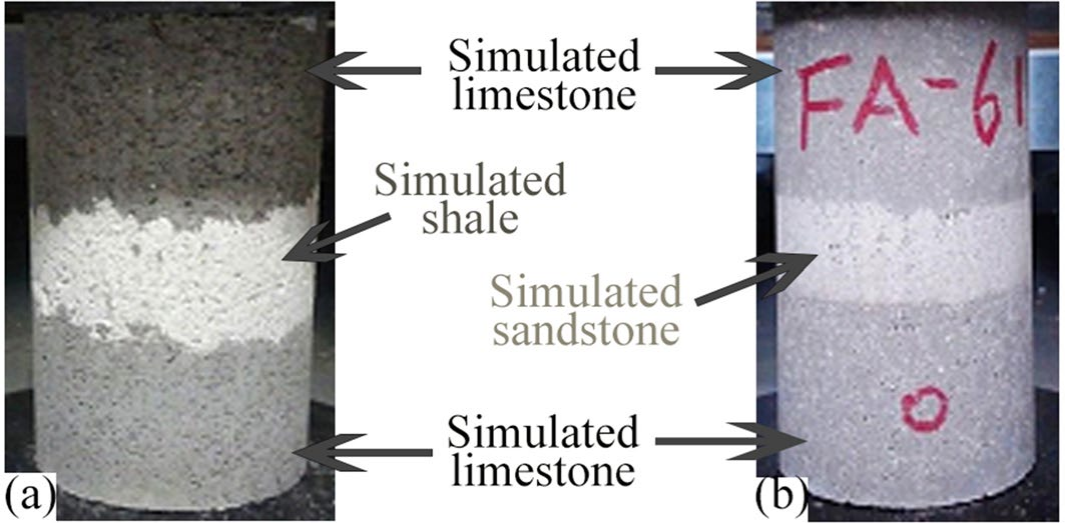
Schematic diagram of (**a**) type I and (**b**) type II specimens.

Two types of simulated composite rock masses with horizontal interlayers were prepared in this study. Type I was a simulated composite rock specimen of limestone interbedded with shale. It was made of simulated shale sandwiched between two layers of simulated limestone on top and bottom. Type II was a simulated composite rock specimen of limestone interbedded with sandstone. It was made of simulated sandstone sandwiched between two layers of simulated limestone on top and bottom. The compressive strength of the interlayer in the type I composite rock specimen was lower than that in type II composite rock specimen. A schematic diagram of type I and type II is shown in Fig. 2. A format code was used to represent the composite rock specimens in this study. Letter “A” and letters “FA” represent the type I and type II composite rock specimens, respectively. Numbers represent the thickness ratio of the main rock mass to the interlayer. For example, “A-51” represents the type I composite rock specimen with a thickness ratio of 1:5 between the interlayer and the main rock mass.

### Uniaxial compression tests

**Figure 3:**
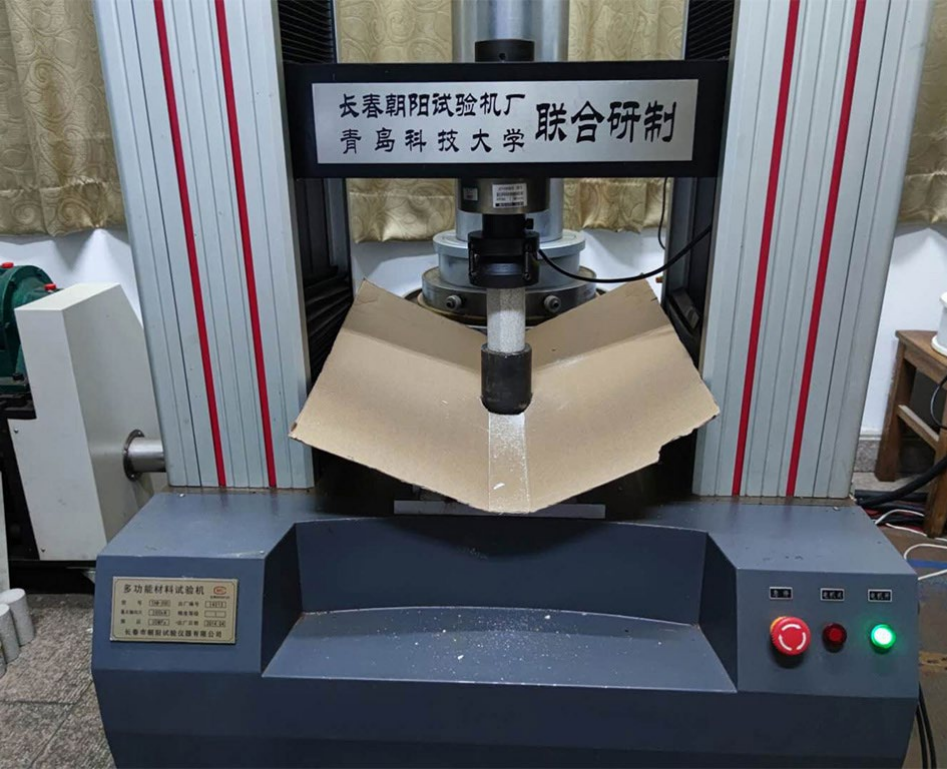
TAW-200 electronic multi-function material mechanics testing machine.

Uniaxial compression tests were carried out based on the guidance of ASTM D2938^30^. A computer-controlled multifunctional material mechanics testing machine was employed to perform the uniaxial compression tests, as shown in Fig. 3. To obtain the full stress–strain curve of the composite rock samples as much as possible, the test was performed in displacement-controlled mode with a loading rate of 0.01 mm/s. Typical stress–strain curves were selected for analysis and average values of the mechanical properties were recorded.

## Mechanical analysis and failure mode prediction

Studying the stress distribution of the composite rock mass was the key to determining the direct cause and mechanism of failure, predicting the failure mode and preventing accidents. This study established a mechanical model of a composite rock mass with a horizontal interlayer under uniaxial compression to conduct mechanical analysis and failure mode prediction. According to previous research, the rock mass as a whole could be regarded as macroscopic and anisotropic, and each rock in the rock mass was treated as a homogeneous continuous isotropic medium^31^.

### Stress state of the interface

**Figure 4:**
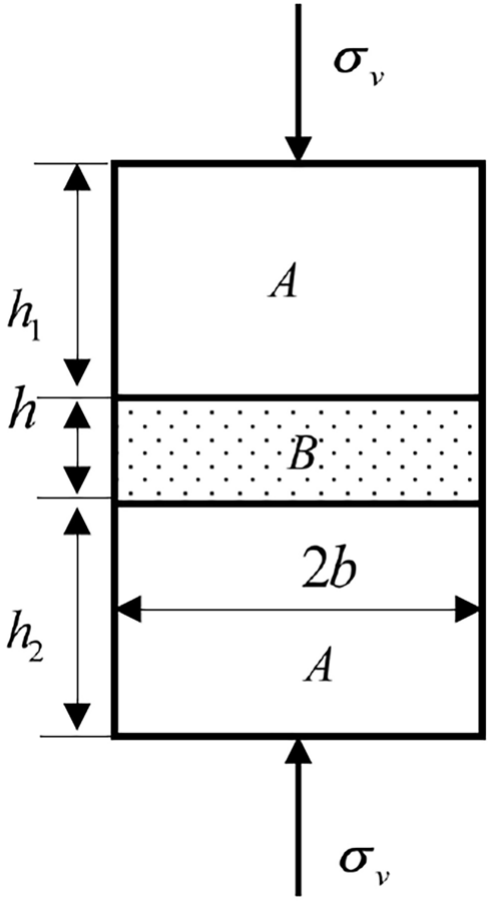
Uniaxial compression mechanical model of composite rock mass specimen with a weak horizontal interlayer.

Based on the theory of elasticity, it was assumed that the rock body as a whole was macroscopically anisotropic and that each rock in the rock body was homogeneous and continuously isotropic, which was considered a planar problem^32^. The uniaxial compression mechanical model of a typical composite rock specimen with a horizontal interlayer is shown in Fig. 4. where *A* is the main rock mass; *B* is the weak interlayer; *h*_1_, *h,* and *h*_2_ represent their thicknesses, respectively; and *b* is the diameter of the specimen. Let *E*_*A*_ and *μ*_*A*_ represent the elastic modulus and Poisson's ratio of the main rock mass, respectively. Let *E*_*B*_ and *μ*_*B*_ represent the elastic modulus and Poisson's ratio of the weak interlayer. Rock mass *A* has higher strength and lower deformability than weak interlayer *B*. Thus, we have the relationship *E*_*A*_ > *E*_*B*_, *μ*_*A*_ < *μ*_*B*_. When there is a vertical stress *σ*_*v*_ along the axis of the column specimen, the rock mass deforms in both the axial and transverse directions. The derived stress will be generated at the interface between rock *A* and *B* due to their different deformability.

**Figure 5:**
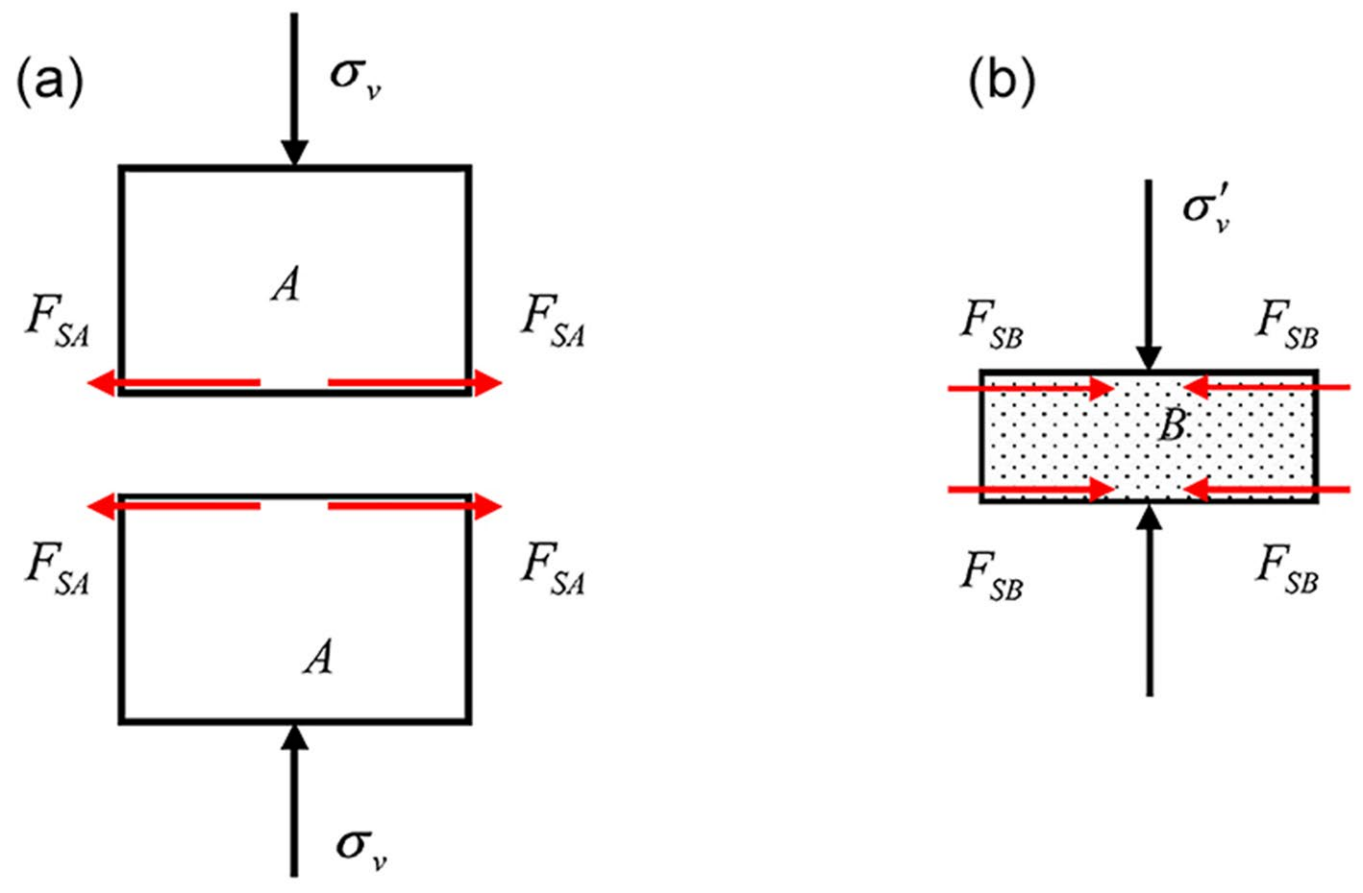
Derived friction binding force distribution of (**a**) the main rock mass and (**b**) the interlayer under uniaxial compression.

Suppose the interface between rock *A* and *B* layers is a structural plane without tensile strength, and there is no other filling between them when there is a vertical stress *σ*_*v*_. In that case, frictional restraint *F*_*S*_ will be produced in the transverse direction. According to the friction law, the value of *F*_*S*_ can be calculated as follows:

1 $$ F_{S} = \sigma_{v} fb $$

where *f* is the coefficient of friction between rock masses *A* and *B*, as shown in Fig. 5. Due to the larger transverse strain of rock *B* compared with *A*, the directions of the friction binding force derived on each rock mass can be found according to Newton's third law.

If the interface between rock *A* and *B* has a strong bonding force, there is no filling between them. When there is a vertical stress *σ*_*v*_, to ensure continuous deformation and coordination, there will be a transverse binding force. The force distribution is similar to that shown in Fig. 5. The value of *F*_*S*_ can be described as follows:

2 $$ F_{S} < \sigma_{v} fb < F_{{S_{\max } }} = \sigma_{v} fb + C. $$

The derived binding force parallel to the interface will be generated when there is a vertical stress. At the same time, due to the emergence of the derived binding force, the stress state at the interface may change from a unidirectional stress state to a bidirectional stress state (a three-directional stress state in space), which may significantly influence the failure mode of the composite rock mass.

### Stress analysis of the weak interlayer and the main rock mass

**Figure 6:**
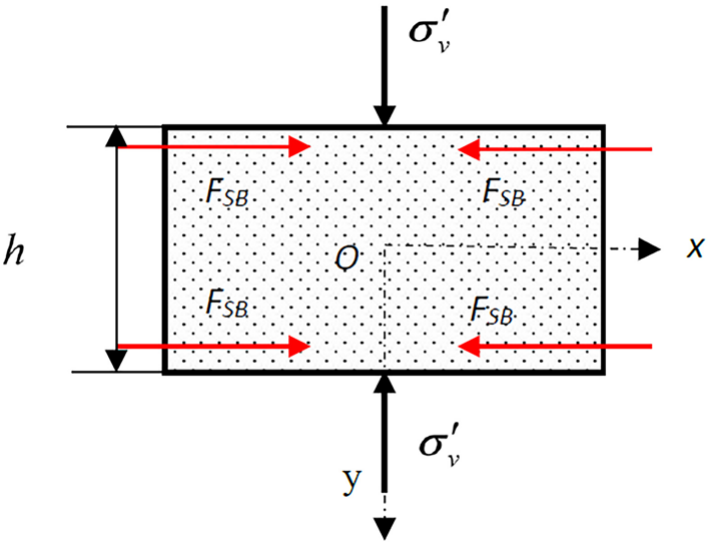
Mechanical model of the weak interlayer under uniaxial axis compression.

The mechanical model of the weak interlayer is shown in Fig. 6. The semi-inverse method of elastic mechanics is used to analytically calculate the stress distribution.

Assuming that the longitudinal stress distribution on all contact surfaces is uniform and does not change along the specimen width, then the axial stress *σ*_*y*_ in the specimen can be regarded as a function of *y*. According to the relationship between the Airy stress function and stress component, when ignoring the weight of the specimen, the axial stress *σ*_*y*_ can be described as follows:

3 $$ \sigma_{y} = \frac{{\partial^{2} \varphi }}{{\partial x^{2} }} = f\left( y \right) $$

where *φ* is the stress function, and the expression for the stress function *φ* can be obtained by integrating Eq. (3):

4 $$ \frac{\partial \varphi }{{\partial x}} = xf\left( y \right) + f_{1} \left( y \right) $$

5 $$ \varphi = \frac{{x^{2} }}{2}f\left( y \right) + xf_{1} \left( y \right) + f_{2} \left( y \right) $$

where *f*_1_(*y*) and *f*_2_(*y*) are functions of the variable *y*. The terms related to the consistency equation are:

6 $$ \left\{ \begin{gathered} \frac{{\partial^{4} \varphi }}{{\partial x^{4} }} = 0 \hfill \\ \frac{{\partial^{4} \varphi }}{{\partial x^{2} \partial y^{2} }} = \frac{{d^{2} f\left( y \right)}}{{dy^{2} }} \hfill \\ \frac{{\partial^{4} \varphi }}{{\partial y^{4} }} = \frac{{x^{2} }}{2}\frac{{d^{4} f\left( y \right)}}{{dy^{4} }} + x\frac{{d^{4} f_{1} \left( y \right)}}{{dy^{4} }} + \frac{{d^{4} f_{2} \left( y \right)}}{{dy^{4} }}. \hfill \\ \end{gathered} \right. $$

Plugging these into the consistency equation ∇^4^*φ* = 0, we can obtain:

7 $$ \left\{ \begin{gathered} \frac{{\partial^{4} \varphi }}{{\partial x^{4} }} + 2\frac{{\partial^{4} \varphi }}{{\partial x^{2} \partial y^{2} }} + \frac{{\partial^{4} \varphi }}{{\partial y^{4} }} = 0 \hfill \\ 0 + 2\frac{{d^{2} f\left( y \right)}}{{dy^{2} }} + \frac{{x^{2} }}{2}\frac{{d^{4} f\left( y \right)}}{{dy^{4} }} + x\frac{{d^{4} f_{1} \left( y \right)}}{{dy^{4} }} + \frac{{d^{4} f_{2} \left( y \right)}}{{dy^{4} }} = 0. \hfill \\ \end{gathered} \right. $$

This formula should be held for all stress fields in the specimen, and *x* can be optionally taken. The Airy stress function can be expressed as follows:

8 $$ \varphi = \frac{{x^{2} }}{2}\left( {Ay^{3} + By^{2} + Cy + D} \right) + x\left( {Ey^{3} + Fy^{2} + Gy} \right) + \left( { - \frac{A}{10}y^{5} - \frac{B}{6}y^{4} + Hy^{3} + Ky^{2} } \right) $$

where *A*, *B*, *C*, *D*, *E*, *F*, *G*, *H*, and *K* are undetermined coefficients. Considering the symmetry of the load and boundary conditions on the end face and free surfaces, we can obtain *A* = *C* = 0, $B = \frac{{{2}F_{S} }}{{b^{{2}} h}}$, $D = - \frac{{F_{S} h}}{{{2}b^{{2}} }} - \sigma^{\prime}_{v}$, $E = F = {0}$, $K = \frac{{F_{S} h}}{{{6}b^{{2}} }} - \frac{{F_{S} }}{h}$, and $H = {0}$. Substituting these values into the stress component derived from Eq. (8), we can obtain the expression of each stress component:

9 $$ \sigma_{x} = \frac{{2F_{S} }}{{b^{2} h}}x^{2} - \frac{{4F_{S} }}{{b^{2} h}}y^{2} + \frac{{F_{S} h}}{{3b^{2} }} - \frac{{2F_{S} }}{h} $$

10 $$ \sigma_{y} = \frac{{2F_{S} }}{{b^{2} h}}y^{2} - \frac{{F_{S} h}}{{2b^{2} }} - \sigma^{\prime}_{v}  $$

11 $$ \tau_{xy} = - \frac{{4F_{S} }}{{b^{2} h}}xy. $$

**Figure 7:**
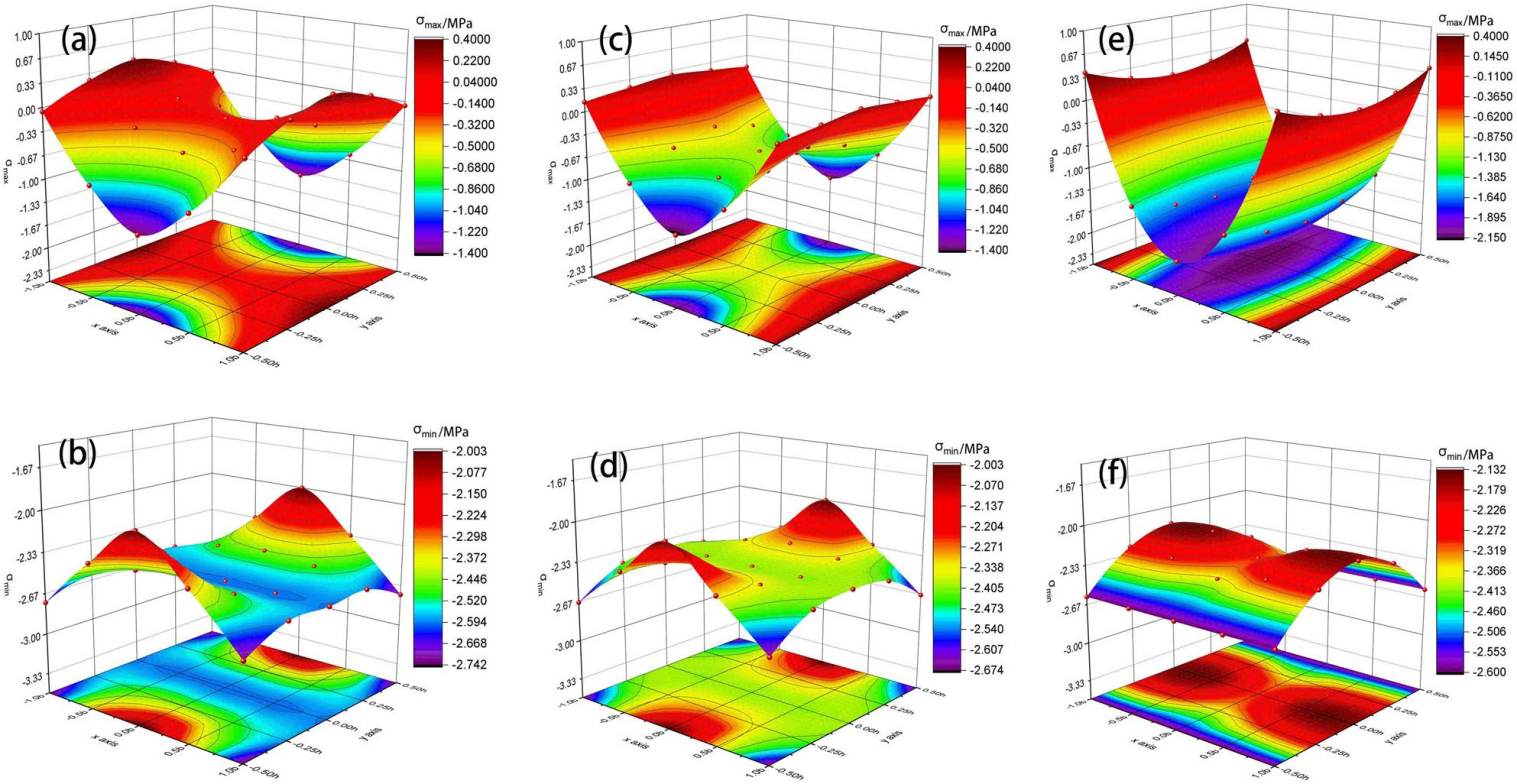
Principal stress distribution of the interlayer when (**a**,**b**): h/H = 1/2, (**c**,**d**): h/H = 1/3, and (**e**,**f**): h/H = 1/8.

The stress distribution of the weak interlayer will be discussed separately according to the thickness of the interlayer. The friction binding force was determined according to the limit condition without distinguishing the adhesion between interfaces. To directly reflect the stress distribution, let the coefficient of friction between rocks be 0.4 and let the axial stress be 2 MPa. To study the stress distribution characteristics of composite rock with a horizontal weak interlayer, different ratios of the interlayer height (h) and the whole rock specimen height (H) were determined to be 1:2, 1:3, and 1:8. According to Eqs. (9), (10), and (11), the principal stress of each point in the weak interlayer can be calculated. Figure 7 shows the principal stress distribution of the interlayer with different thickness ratios. The maximum principal stress distribution in the interlayer are shown Fig. 7a,c,d. The minimum principal stress distribution in the interlayer are shown in Fig. 7b,d,f. It can be seen that the minimum principal stress is always the compressive stress regardless of the thickness of the interlayer. The extremum principal tensile stress of the thick interlayer is greater than that of the thin interlayer. When the interlayer is relatively thicker (h/H = 1/2), the interface is in a bidirectional compression state. When the interlayer is relatively thinner (h/H = 1/8 or h/H = 1/3), the interface is in a bidirectional tension–compression state.

**Figure 8:**
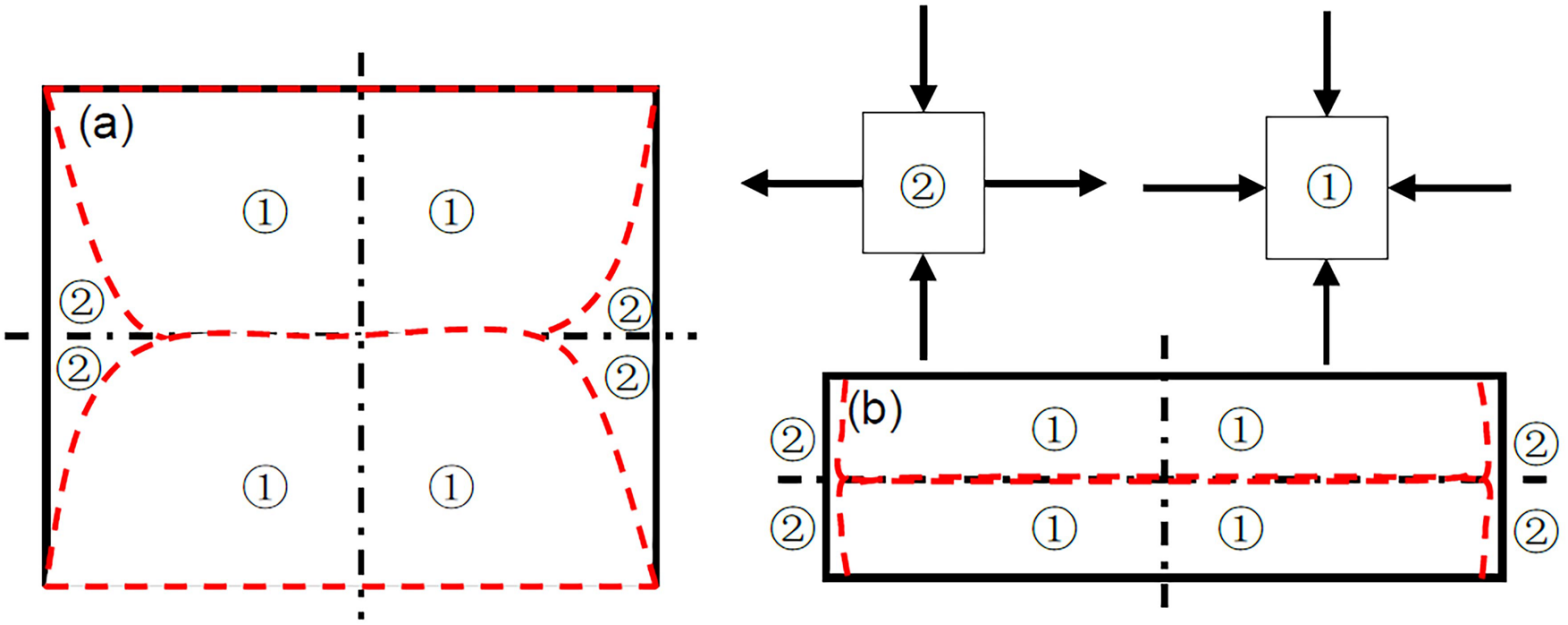
Stress states of (**a**) thick interlayer and (**b**) thin interlayer.

Based on Fig. 7, positive and negative cases of principal stresses at any location in the interlayer for a certain interlayer thickness can be found. If both the maximum principal stress and the minimum principal stress were negative, then the location was compressed in both directions. If the maximum and minimum principal stresses were negative and positive, the location was under tension and compression in both directions. Accordingly, Fig. 8 shows the stress states of thick interlayer and thin interlayer. The red line represents the boundary of different force states, separating the two-way compressed region and the two-way tensioned compressed region. This may help determine the tensile and compression areas with their boundaries and predict the failure mode of the interlayer. The failure of the interlayer is related to the thickness ratio, and the failure of a thick interlayer more likely easier than that of a thin interlayer. When the interlayer is thick, the failure mode is a symmetric cone, and the failure will start from the middle of the specimen and then peel off and extend to the interfaces on both sides. When the interlayer is thin, it will first crack from the interface and then extend from the two sides to the middle, and the outer surface of the interlayer expands or peels out in an approximate ring.

**Figure 9:**
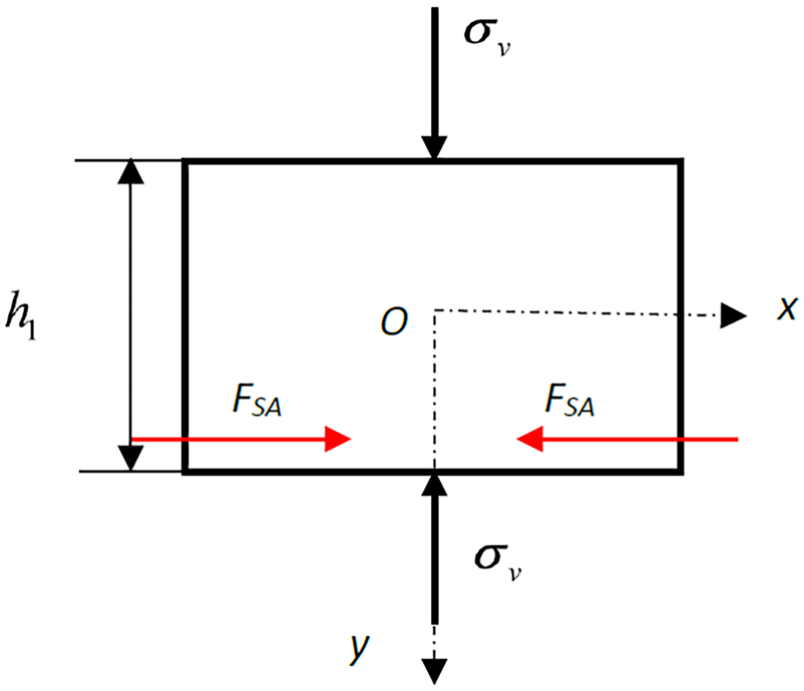
Mechanical model of the main rock mass.

Figure 9 shows a mechanical model of the main rock mass. The analytical stress distribution of the main rock mass can be calculated based on the semi-inverse method. The analysis process of the main rock mass is similar to that of the interlayer. The expression of each stress component can be expressed as:

12 $$ \sigma_{x} = - \frac{{x^{2} }}{2}\left( {\frac{{12F_{S} }}{{h_{1}^{2} b^{2} }}y + \frac{{2F_{S} }}{{h_{1} b^{2} }}} \right) + \frac{{4F_{S} }}{{h_{1}^{2} b^{2} }}y^{3} + \frac{{2F_{S} }}{{h_{1} b^{2} }}y^{2} + \left( {\frac{{6F_{S} }}{{h_{1}^{2} }} - \frac{{3F_{S} }}{{5b^{2} }}} \right)y + \left( { - \frac{{F_{S} h_{1} }}{{6b^{2} }} + \frac{{F_{S} }}{{h_{1} }}} \right) $$

13 $$ \sigma_{y} = - \frac{{2F_{S} }}{{h_{1}^{2} b^{2} }}y^{3} - \frac{{F_{S} }}{{h_{1} b^{2} }}y^{2} + \frac{{F_{S} }}{{2b^{2} }}y + \frac{{F_{S} h_{1} }}{{4b^{2} }} - \sigma_{v}  $$

14 $$ \tau_{xy} = - x\left( { - \frac{{6F_{S} }}{{h_{1}^{2} b^{2} }}y^{2} - \frac{{2F_{S} }}{{h_{1} b^{2} }}y + \frac{{F_{S} }}{{2b^{2} }}} \right). $$

**Figure 10:**
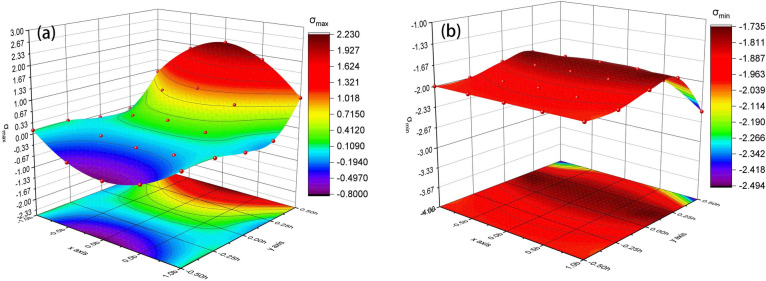
(**a**) Maximum principal stress and (**b**) minimum principal stress distribution of the main rock mass with the middle thick interlayer.

To contrast with the stress distribution of the interlayer, the stress distribution of the main rock mass is assessed with a middle thick interlayer (i.e., h/H = 1/3). The principal stress distribution of the main rock mass is shown in Fig. 10. The principal stress combination at the interface of the main rock mass is all bidirectional tension–compression stress at each point, and its value is higher than that of the interface outside. Therefore, it can be inferred that the failure of the main rock mass would start from the interface. The cracks would develop in the centre of the interface and expand outwards with a tearing form. The large tensile failure would lead to a failure of the interlayer. It is possible that the interlayer might have been damaged before the failure of the main rock mass occurred due to the high strength of the main rock mass.

In general, due to the difference in the mechanical properties of the interlayer and the main rock mass on both sides of the interface, the derived friction constraints will be generated in the composite rock under uniaxial compression. The derived friction constraint does not cause material shear failure along the interface for the interface with cohesive force. Still, the interface and the stress state outside the interface could change and then affect the failure mode due to the existence of derived friction. Therefore, there are four possible failure modes of composite rock mass specimens under uniaxial compression.

**Figure 11:**
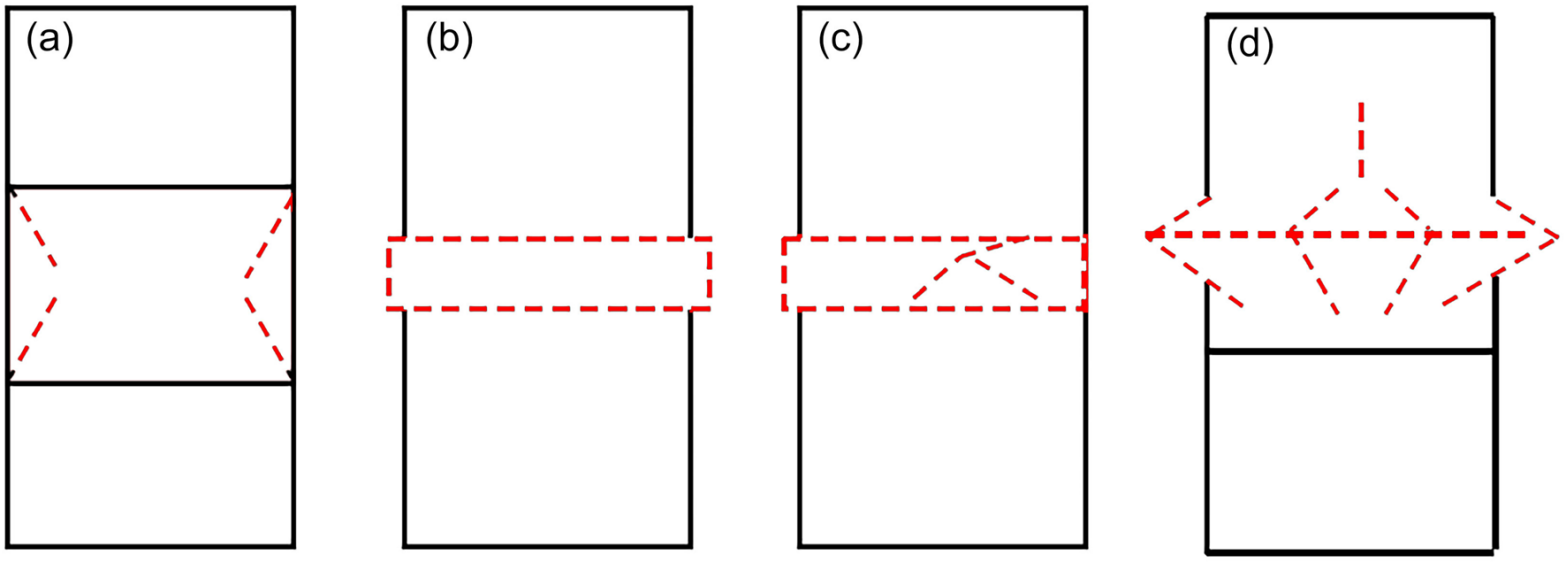
Possible failure modes of composite rock mass specimens with horizontal interlayer under uniaxial compression.

The possible failure modes are summarized in Fig. 11. The red line represents the cracks and deformation of the interlayer after failure. The symmetrical conical failure of the thick interlayer shown in Fig. 11a was inferred from Fig. 8a. The boundary of different force states in Fig. 8a coincided with the red line in Fig. 11a. During the failure process, spalling may occur along the cracks in the bidirectional tensile and compressive stress regions on the outside of the cone due to the presence of tensile forces, which echoes the results of the principal stress distribution derived from Eqs. (9), (10), and (11). This failure mode may occur when the interlayer is thick, and the strength of the interlayer is significantly weaker than that of the main rock mass. According to Fig. 11b, when the interlayer was thin and the strength of the interlayer was significantly weaker than that of the main rock mass, uniform annular expansion failure modes may have occurred, as shown in Fig. 11b. Considering a practical situation in which the interlayer material may not be uniform and continuous, there may be an uneven annular expansion accompanied by cracks, as shown in Fig. 11c. When the difference in strength between the interlayer and the main rock mass was not very large, the failure mode may have occurred as shown in Fig. 11d.

## Experimental results and discussion

**Figure 12:**
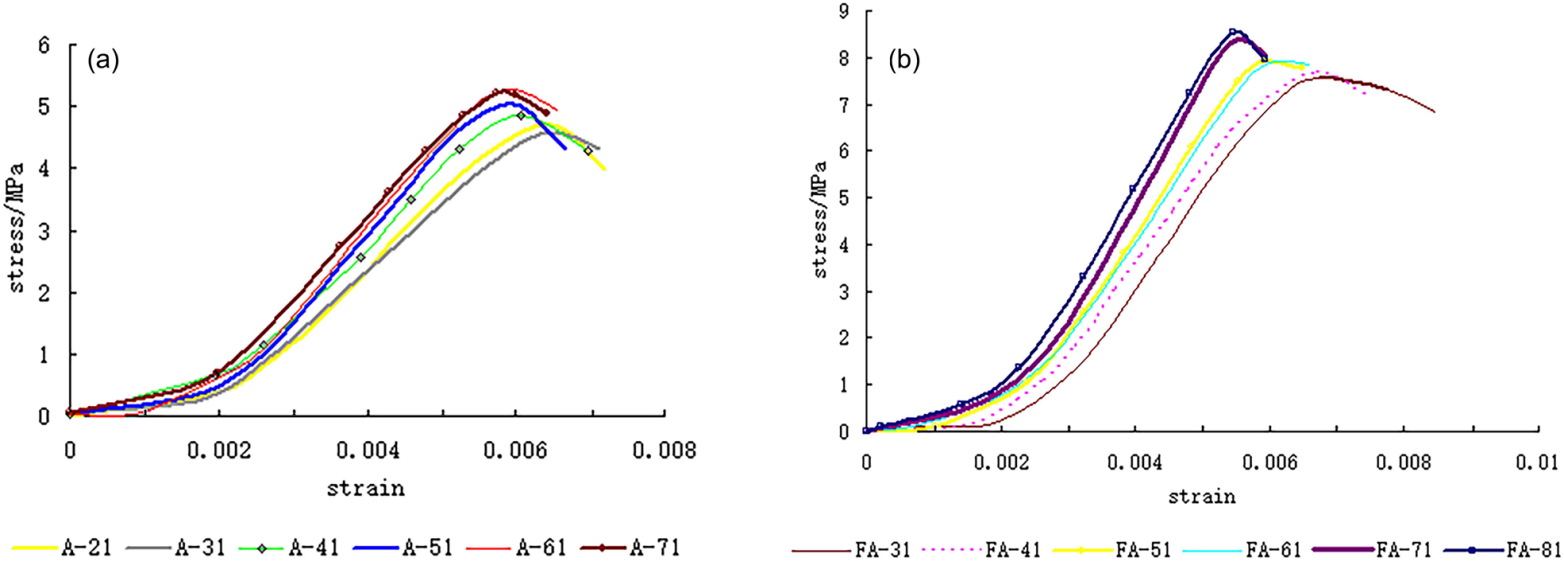
Stress–strain curves of (**a**) type I and (**b**) type II composite rock samples.

The experimental stress–strain curves of the two types of composite rock with horizontal interlayers of different thicknesses are shown in Fig. 12. The stress–strain curves of the two types of composite rock masses are similar. There are pore compaction stages, elastic stages, plastic stages, and strain softening stages in each stress–strain curve. It can also be seen that the peak strain increased with increasing thickness ratio. This may be due to the larger deformation capacity of the interlayer.

**Figure 13:**
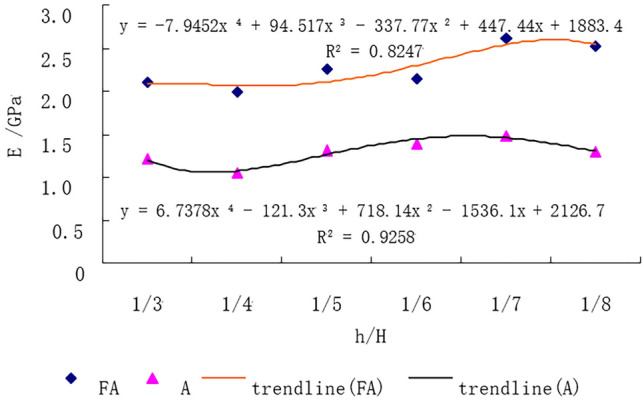
Elastic modulus of composite rock specimens.

The elastic modulus of the composite rock mass with horizontal interlayers of different thicknesses is shown in Fig. 13. The elastic modulus of type II composite rock mass is higher than that of the type I composite rock mass, which may be due to the differences in interlayer materials. Although there are fluctuations in the modulus of elasticity, it can be inferred that the elastic modulus increases with decreasing thickness ratio. These results were related to the difference in mechanical parameters of rock-like materials used to simulate composite rocks.

**Figure 14:**
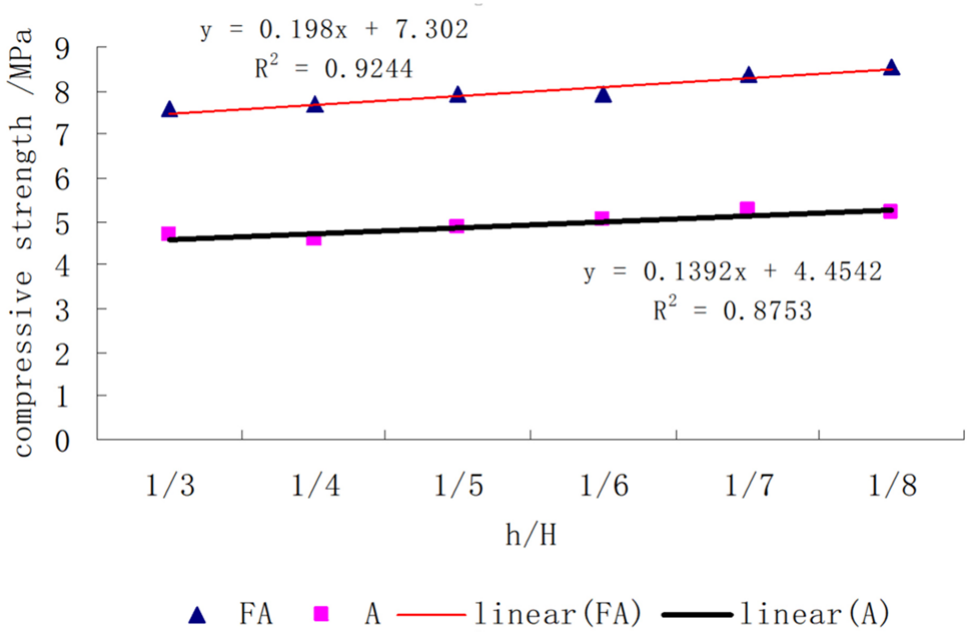
Uniaxial compressive strength of composite rock specimens.

The uniaxial compressive strength of the composite rock mass with horizontal interlayers of different thicknesses is shown in Fig. 14. The compressive strength increased linearly with decreasing thickness ratio. This result is consistent with a previous experimental investigation on sandstone with a siltstone interlayer^33^ and echoes the analysis result in “Mechanical analysis and failure mode prediction”. It can also be seen that the compressive strength of the type II composite rock mass is higher than that of the type I composite rock mass, which may be attributable to the higher strength of the interlayer material of type II.

**Figure 15:**
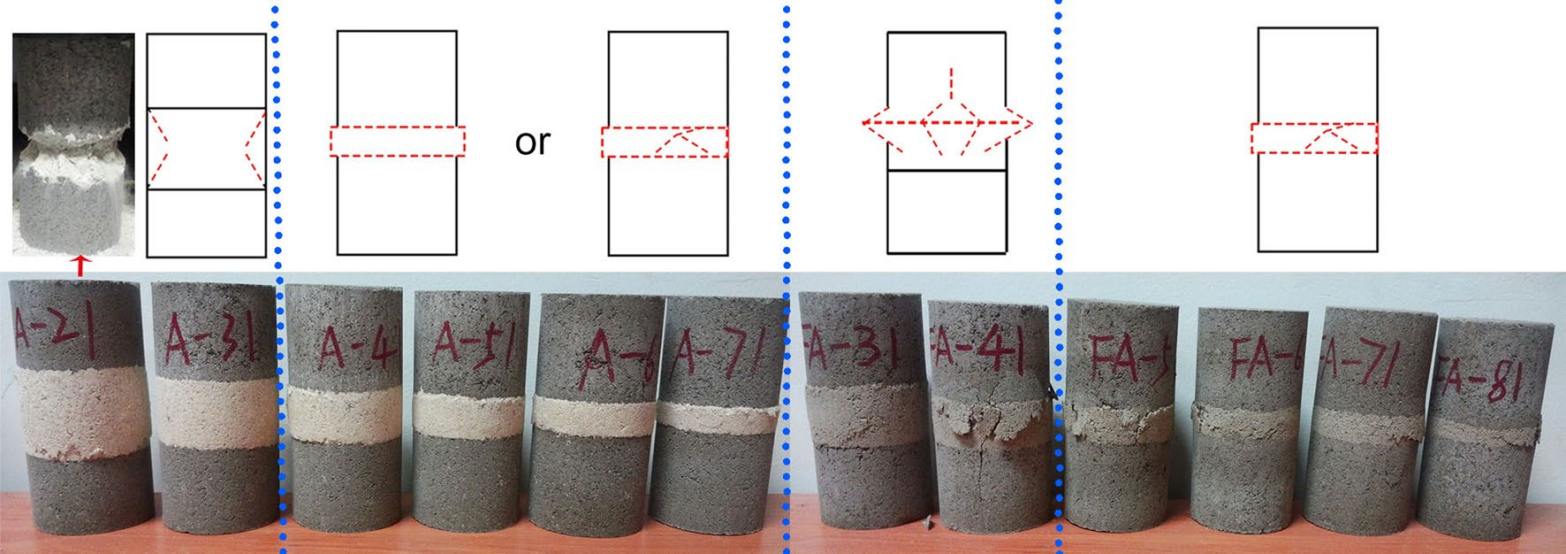
Uniaxial compression failure modes of (**a**) type I and (**b**) type II composite rock specimens.

Figure 15 shows the predicted failure modes and experimental failure modes of the composite rock specimens. The blue dashed line delineates the four different failure modes of the experimental results corresponding to the predicted failure modes in “Mechanical analysis and failure mode prediction”. For type I composite rock specimens, expansion of the weak interlayer can be clearly observed. At the outer edge of the bedding interface, separation between the interlayer and the main rock mass can be seen. When the interlayer is relatively thick (e.g., h/H = 1/3), the failure mode of the interlayer is similar to a drum, and symmetrical conical failure appears as the load continues, which is consistent with Fig. 11a. The main rock mass stays relatively intact, which may be because of the much higher strength of the main rock mass compared with that of the interlayer. When the interlayer is relatively thin (e.g., h/H = 1/7), the failure mode of the interlayer is uniform annular expansion failure or uneven annular expansion failure, which is consistent with Fig. 11b,c. For type II composite rock specimens, the cracks were mainly concentrated in the interlayer and slightly expanded to the main rock mass when the interlayer was relatively thick, which was consistent with Fig. 11d. This may be due to the relatively small difference in strength between the interlayer and main rock mass. When the interlayer was relatively thin, the interlayer expanded unevenly with cracks appearing, which was consistent with Fig. 11c, although the interlayer expansion was much milder, and the cracks in the interlayer were more pronounced for type II specimens. Therefore, it can be inferred that the failure modes of the composite rock were related to both the internal stress distribution under compression and the difference in strength and deformation capacity between the constituent parts of the composite rock. However, for both types of composite rocks, the interface between the interlayer and main rock mass was the weakest link in the failure process because cracks often started from one point at the edge of the interface during compression failure. In practical engineering, the focus should be on the interface between the interlayer and the main rock mass. When the difference in strength between the interlayer and the main rock mass was relatively small and the interlayer was relatively thicker, it was important to pay attention to the transmission of cracks from the interface to the main rock mass for targeted support or warning.

## Conclusions

A mechanical model of a composite rock mass containing a horizontal weak interlayer under uniaxial compression was developed. An analytical solution of the stress component was obtained. It was concluded that a composite rock with a thick interlayer failed more easily than a composite rock with a thin interlayer. The composite rock with a horizontal interlayer had four potential failure modes under uniaxial compression: symmetrical conical failure of the thick interlayer, uniform annular failure of the thin interlayer, mixed failure of the interlayer starting from the interface, and tensile failure of both the interlayer and main rock mass starting from the interface.

Uniaxial compression tests were carried out for two types of composite rock with a horizontal interlayer. It was found that the unconfined compressive strength and modulus of elasticity decreased as the thickness of the interlayer increased. However, the two types of composite rock specimens with the same thickness exhibited different failure modes. The failure mode of composite rock was related to both the internal stress distribution under compression and the difference in deformation capacity and strength between the constituent components. The failure modes in the experiments are all within the predicted range of the failure modes inferred from the mechanical model analysis.

These results verified the validity of the mechanical analysis results and improved the understanding of the mechanical properties of composite rock with a horizontal interlayer. However, simulated rocks rather than real rocks were used in this paper. Although the mechanical properties of both are similar, many natural rock bodies containing various intercalation features should be obtained in the future to conduct experiments under the same conditions to verify the results obtained with artificial composite rock bodies.
